# A Convenient and Safe *O*-Methylation of Flavonoids with Dimethyl Carbonate (DMC) 

**DOI:** 10.3390/molecules16021418

**Published:** 2011-02-09

**Authors:** Roberta Bernini, Fernanda Crisante, Maria Cristina Ginnasi

**Affiliations:** Dipartimento di Agrobiologia e Agrochimica, Università degli Studi della Tuscia, Via S. Camillo De Lellis, 01100 Viterbo, Italy

**Keywords:** methylated flavonoids, ecofriendly methylation reaction, dimethyl carbonate (DMC), green chemistry

## Abstract

Dietary flavonoids exhibit beneficial health effects. Several epidemiological studies have focused on their biological activities, including antioxidant, antibacterial, antiviral, anti-inflammatory and cardiovascular properties. More recently, these compounds have shown to be promising cancer chemopreventive agents in cell culture studies. In particular, *O*-methylated flavonoids exhibited a superior anticancer activity than the corresponding hydroxylated derivatives being more resistant to the hepatic metabolism and showing a higher intestinal absorption. In this communication we describe a convenient and efficient procedure in order to prepare a large panel of mono- and dimethylated flavonoids by using dimethyl carbonate (DMC), an ecofriendly and non toxic chemical, which plays the role of both solvent and reagent. In order to promote the methylation reaction under mild and practical conditions, 1,8-diazabicyclo[5.4.0]undec-7-ene (DBU) was added in the solution; methylated flavonoids were isolated in high yields and with a high degree of purity. This methylation protocol avoids the use of hazardous and high toxic reagents (diazomethane, dimethyl sulfate, methyl iodide).

## 1. Introduction

Flavonoids are a group of low-molecular-weight polyphenolic compounds that occur ubiquitously in all plants, where they play a protective role against predators, pathogens and UV radiations [1]. Being present in fruits, vegetables and beverages, they are integral part of the human diet. In the last few years, dietary flavonoids have gained considerable interest because of their potential beneficial on human health. Experimental studies demonstrated that they show several biological activities including radical scavenging, anti-inflammatory, antimutagenic, anti-HIV, anti-allergic, anti-platelet and antioxidant activities [2]. More recently, the potential utility of these compounds in chemoprevention of cancer has been investigated. Promising biological effects have been revealed in cell culture studies; however, when experiments have been extended to the *in vivo* experiments, in particular, in humans, these results have not been confirmed always. The data have been related to very low oral bioavailability of dietary flavonoids, strictly dependent on the presence of free hydroxyl groups responsible for their susceptibility to glucuronidation, sulfation and oxidation reactions in the intestine and liver that avoids them to pass intact into the systemic circulation [3]. In contrast, methylated flavones were much metabolically stable and showed higher intestinal absorption through human colon adenocarcinoma (Caco-2) cell monolayers compared with their unmethylated analog derivatives, suggesting that methylation protects these compounds from hepatic metabolism [4]. As examples, 7-hydroxyflavone; 7,4’-dihydroxyflavone; 5,7-dihydroxyflavone (chrysin) were undetectable in tissue levels after administration to rats whereas the corresponding methylated derivatives reached high tissue levels [5,6]. Mono and dimethylated flavones showed potent antiproliferative activities [7,8]; they inhibit the carcinogenic-activating cytochrome P450 (CYP) transcription and activities [9], the benzo[a]pyrene activating enzymes and DNA binding in human bronchial epithelial BEAS-2B cells [10], the aromatase, an important target in hormone-sensitive cancers [11]. Furthermore, methylated flavonoids showed effects on multidrug resistance proteins (MRPs), transport proteins which play a central role in the defence of organism against toxic compounds [12]; they exhibit fungicidal properties [13].

**Scheme 1:**
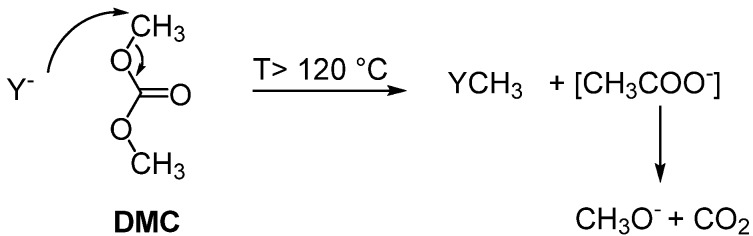
Mechanism of methylation reaction with dimethyl carbonate (DMC).

The *O-*methylation of flavonoids is a common xenobiotic transformation occurring in plants, microbes and mammalians from high selective enzymatic systems, the *O-*methyl transferases [14]. Chemically, the reaction was usually performed treating the phenolic compounds with hazardous and high toxic reagents such as diazomethane, dimethyl sulfate and methyl iodide. In addition, their use requires stoichiometric amount of strong bases to neutralize acidic by-products resulting in the production large quantities of inorganic salts that need appropriate and expensive disposal. In the last few years, dimethyl carbonate (DMC), a non toxic compound, has emerged as a safe and efficient alternative reagent for ecofriendly methylation reactions. At high temperatures (T > 120 °C, usually 160 °C) and in the presence of a nucleophile (ArOH, ArSH), the electrophilic methyl group of DMC was attacked producing and the corresponding methylated compound was isolated [15]; the only by-products were methanol and CO_2_ (Scheme 1). In consideration of the high temperatures required, the reaction was generally performed under gas liquid phase-transfer catalysis (GL-PTC) conditions or in autoclave over alkali ion-exchanged zeolites, alumina, alumina-silica, CrPO_4_, AlPO_4_, CrPO_4_-AlPO_4_, AlPO_4_-Al_2_O_3_, cesium carbonate [16,17,18,19,20,21]. The search for milder and more practical conditions to carry out the methylation reactions with DMC is an interesting topic.

## 2. Results and Discussion

**Scheme 2:**
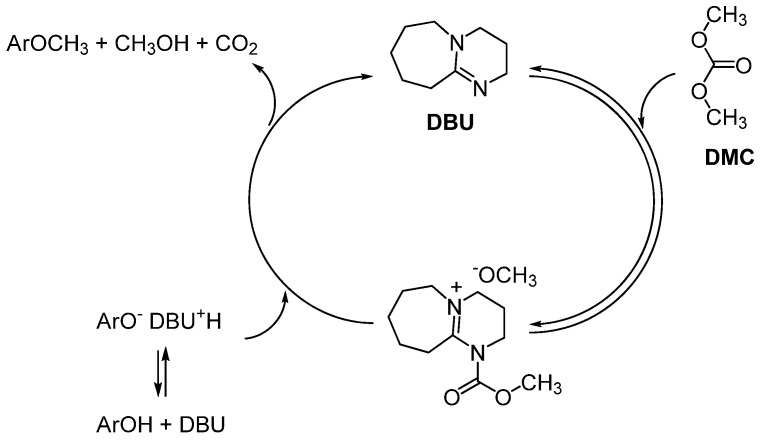
Plausible role of DBU in phenol methylation with DMC.

In consideration of the biological activities of methylated flavonoids as well as of the ecofriendly properties of DMC, we optimized the experimental conditions for a convenient and safe *O-*methylation of a large panel of flavonoids. In order to provide a practical methylation protocol in DMC, we added 1,8-diazabicyclo[5.4.0]undec-7-ene (DBU) in the solution, already utilized in the methylation of phenols, indoles and benzimidazoles [22]. According to the proposed mechanism, DBU performs as a nucleophilic catalyst and reacts with DMC to generate a more active methylating agent which presumably reduces the activation energy required for the methylation reaction (Scheme 2). 

**Scheme 3:**
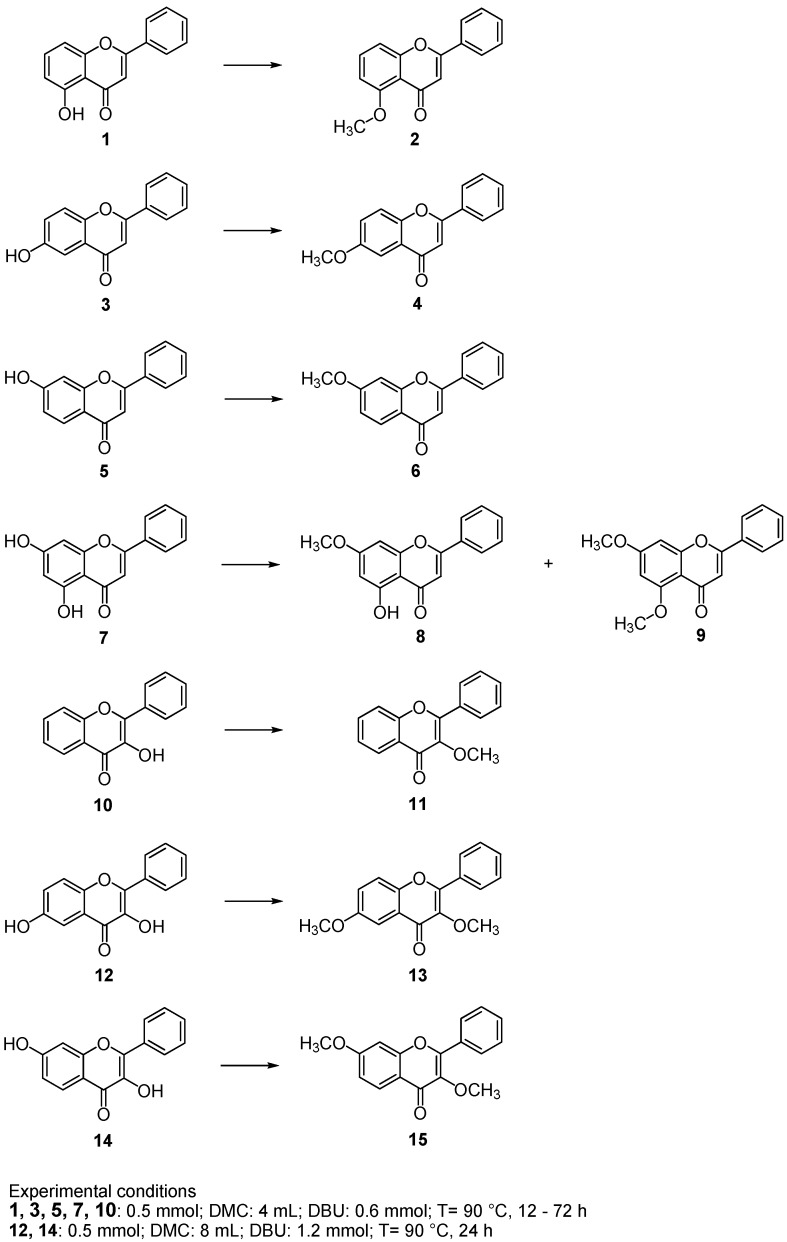
Methylation reaction of flavonoids with DMC/DBU system.

Mono and dihydroxylated flavones **1**, **3**, **5**, **7** and flavonols **10**, **12**, **14** were the starting substrates (Scheme 3). *O-*methylation reaction was performed at reflux temperature (90 °C) using a large excess of DMC and DBU in a stoichiometric amount respect to flavonoid. As reported in Table 1, flavonoids were converted into the corresponding methylated compounds in 12–72 h depending on the substrate. To the best of our knowledge, this is the first paper dealing the *O-*methylation of flavonoids under mild experimental conditions by using DMC. 5-Methoxyflavone (**2**) was isolated after 48 h (Table 1, entry 1), 6-methoxyflavone (**4**) after 36 h and 7-methoxyflavone (**6**) after only 12 h (Table 1, entries 2 and 3). In all cases quantitative yields of methylated products were obtained. The slow reactivity of 5-hydroxy-flavone (**1**) may be due to the well known hydrogen bonding of 5-OH group with carbonyl group [23]. The methylation of 5,7-dihydroxyflavone (**7**) afforded to 5-hydroxy-7-methoxy derivative (**8**) and 5,7-dimethoxyflavone (**9**) after 48 h, being **8** was the main compound isolated (Table 1, entry 4); by prolonging the reaction time until 72 h we obtained a quantitative yield of **9** (Table 1, entry 5). As expected, on the basis of the different reactivity of the 5-OH and 7-OH group, we did not observe the formation of the 5-methoxy-7-hydroxy derivative. In a similar way, we performed the *O-*methylation of flavonols **10**, **12**, **14** (Table 1, entries 6–8). 3-Methoxyflavone derivatives **11**, **13** and **15** were isolated in quantitative yield after 24 h. 

Flavanols **12** and **14** were barely soluble in DMC, so a double quantity of reagents was added in order to obtain a clear solution. The lack of selectivity in the methylation of the phenolic and alcoholic group in C-3 was probably due to this excess of reagents. It is known in the literature that 3-methoxyflavone derivatives showed high antioxidant activity [24], exhibited potent neuroprotective effects on the oxidative injuries to neuronal cells [25]. Finally, the methylation reaction was extended to several polyhydroxylated flavonoids. Unfortunately, these compounds showed a low solubility in DMC/DBU system also adding an excess of reagents. As a consequence, we observed a poor reactivity. As example, 3,5,7,3’,4’-pentamethylated flavone (methylated quercetin) was isolated in only 28% after a long reaction time (72 h).

**Table 1:** Experimental data of methylation reaction of flavonoids depicted in Scheme 2.

Entry	Substrate	Time (h)	Conv. (%)	Yield (%)
1	**1 **	48	> 98	**2** : >98
2	**3 **	36	> 98	**4** : >98
3	**5 **	12	> 98	**6** : >98
4	**7**	48	>98	**8** : 70; **9**: 30
5	**7**	72	>98	**9**: 95
6	**10 **	24	> 98	**11**: >98
7	**12 **	24	> 98	**13**: >98
8	**14 **	24	> 98	**15**: >98

## 3. Experimental

### 3.1. Materials and methods

All chemicals were purchased from Sigma-Aldrich and were of analytical grade. Silica gel 60 F254 plates and silica gel 60 were obtained from Merck. ^1^H-NMR and ^13^C-NMR were recorded on a Bruker 200 MHz spectrometer using CDCl_3_ as solvent. All chemical shifts are expressed in ppm (δ scale) and coupling constants in Hz. GC-MS analysis was performed on a Shimadzu VG 70/250S apparatus equipped with a CP-SIL 8 CB-MS column (25 m, 0.25 mm and 0.25 mm film thickness). The analyses were performed using an isothermal temperature profile of 100 °C for 2 minutes, followed by a 10 °C/min temperature gradient for 15 minutes until 280 °C. The injector temperature was 280 °C.

### 3.2. General procedure for the methylation reaction of flavonoids

In a typical experiment, the substrate (0.5 mmol) was dissolved in dimethyl carbonate (4 mL); then DBU (0.6 mmol) was added. The solution was kept at T = 90 °C under magnetic stirring and monitored by TLC. After disappearance of the substrate, the solvent was evaporated under reduced pressure in the presence of methanol (3 mL) as an azeotropic mixture. The residue was solubilised with ethyl acetate (10 mL) and treated with a solution of 1N HCl (5 mL). The final products were extracted with ethyl acetate (3 × 10 mL); the reunited organic extracts were washed with a saturated solution of NaCl and dried over Na_2_SO_4_. After filtration and evaporation of the solvent, methylated compounds were purified by chromatography on column by using silica gel (230–400 mesh) and the mixture CH_2_Cl_2_/CH_3_OH = 9.8/0.2 as eluent. Analytical and spectroscopic data were recorded.

*5-Methoxyflavone* (**1**). White solid, quantitative yield. Mp 130–133 °C (lit.[26] 131 °C). Spectroscopic data were according to the literature [27].

*6-Methoxyflavone* (**3**). Yellow oil, quantitative yield. Mp 164 °C (lit.[26] 161–163 °C). Spectroscopic data were according to the literature [27]. 

*7-Methoxyflavone* (**5**). White solid, quantitative yield. Mp 108–110 °C (lit.[28] 109–110 °C). Spectroscopic data were according to the literature [28].

*5-Hydroxy-7-methoxyflavone* (**8**). White solid, 70% yield. Mp 164–166 °C (lit. [29] 167 °C). Spectroscopic data were according to the literature [29].

*5,7-Dimethoxyflavone* (**9**). White solid, quantitative yield. Mp 200–203 °C (lit. [29] 202 °C). Spectroscopic data were according to the literature [29]. 

*3-Methoxyflavone* (**11**). Colorless oil, quantitative yield. Spectroscopic data were according to the literature [30].

*3,6-Dimethoxyflavone* (**13**). Colorless oil, quantitative yield. ^1^H-NMR (CDCl_3_) δ 3.87 (s, 3H, OCH_3_), 3.89 (s, 3H, OCH_3_), 7.27 (m, 1H, H-7), 7.47 (m, 4H, H-8, 3’, 4’, 5’), 7.590 (d, *J* = 3.0, 1H, H-5), 8.07 (m, 2H, H-2’, 6’). ^13^C-NMR (CDCl_3_) δ 55.9, 60.1, 104.5, 119.4, 123.9, 124.8, 128.4, 128.5, 130.6, 131.0, 141.1, 150.2, 155.4, 156.6, 174.9. GC-MS (*m/z*): 282, 150, 105, 89, 77, 63, 51. 

*3,7-Dimethoxyflavone* (**15**). Colorless oil, quantitative yield. ^1^H-NMR (CDCl_3_) δ 3.86 (s, 3H, OCH_3_), 3.89 (s, 3H, OCH_3_), 6.95 (m, 2H, H-6, 8), 7.45 (m, 3H, H-3’, 4’, 5’), 8.00 (m, 3H, H-5, 2’, 6’). ^13^C-NMR (CDCl_3_) δ 55.8, 60.1, 99.9, 114.4, 119.0, 127.1, 128.3, 128.4, 128.5, 128.6, 130.5, 131.0, 141.5, 155.2, 158.0, 164.0, 175. GC-MS (*m/z*): 282, 150, 105, 89, 77, 63, 51. GC-MS (m/z): 282, 150, 105, 89, 77, 63, 51.

## 4. Conclusions

Mono- and dimethylated flavonoids were prepared by using dimethyl carbonate (DMC) both as solvent and reagent in the presence of 1,8-diazabicyclo[5.4.0]undec-7-ene (DBU) to promote the methylation reaction under mild and practical conditions. Final products were isolated in high yields and high degree of purity. To the best of our knowledge, this is the first paper dealing the *O-*methylation of flavonoids using non toxic reagents in order to obtain the corresponding methylated derivatives, bioactive compounds potentially useful in cancer therapy.
